# Antisense‐mediated APP suppression as potential therapeutic for Down Syndrome Alzheimer’s Disease

**DOI:** 10.1002/alz.092628

**Published:** 2025-01-09

**Authors:** Hien T Zhao, Xu‐Qiao Chen, Utpal Das, Xinxin Zuo, Brittany T Ford, Holly B Kordasiewicz, William C. Mobley

**Affiliations:** ^1^ Ionis Pharmaceuticals, Inc., Carlsbad, CA USA; ^2^ University of California San Diego, La Jolla, CA USA; ^3^ Department of Neurosciences, University of California, San Diego, La Jolla, CA USA

## Abstract

**Background:**

Increased APP gene dosage is both necessary and sufficient to result in Down Syndrome Alzheimer’s Disease (DSAD) in humans and AD‐related degenerative changes in mouse models of DS.

**Method:**

We tested antisense oligonucleotides (ASOs) designed to suppress APP expression via RNAseH1‐mediated degradation in the Dp(16)1Yey or Dp(16) model of Down Syndrome. Dp(16) is trisomic for human chromosome 21 syntenic regions on murine chromosome 16, containing 115 genes including APP. To evaluate efficacy of APP suppression, Dp16 and 2N euploid mice at 6‐8 months of age were treated with a mouse App ASO, and endosomal pathology and downstream pathological processes were evaluated.

**Result:**

APP suppression reverses not only Rab5 hyperactivation in this model, but also abnormal hyperactivation of other Rab GTPases such as Rab7 and Rab11. Furthermore, tau hyperphosphorylation was ameliorated, and neurotrophin signaling was restored following APP suppression.

**Conclusion:**

Taken together, these data support the hypothesis that APP suppression should restore normal endosomal function and neurotrophic signaling and benefit disease, and the potential of antisense‐mediated APP suppression as a disease‐modifying therapy for DSAD.

